# Supplementary material for: MetaQTL: a package of new computational methods for the meta-analysis of QTL mapping experiments
Source: BMC Bioinformatics. 2007 Feb 8;8:49. doi: 10.1186/1471-2105-8-49 (PMC1808479; doi:10.1186/1471-2105-8-49)
Supplement: Additional File 7 — MetaQTL Package : jar file and tutorial. This Zip archive contains both the MetaQTL JAR file and the files of the tutorial. [file 1471-2105-8-49-S7.zip › org.inra.metaqtl/doc/org/metaqtl/factory/EMResultFactory.html]

EMResultFactory


|  |  |  |
| --- | --- | --- |
| **Overview** \| **Package** \| **Class** \| **Use** \| **Tree** \| **Deprecated** \| **Index** \| **Help** | |  |
| **PREV CLASS**   **NEXT CLASS** | **FRAMES**    **NO FRAMES**     **All Classes** |  |
| SUMMARY: NESTED \| FIELD \| CONSTR \| METHOD | DETAIL: FIELD \| CONSTR \| METHOD |  |


---


## org.metaqtl.factory Class EMResultFactory

```
java.lang.Object
  org.metaqtl.factory.EMResultFactory
```

---

``` public class EMResultFactory extends java.lang.Object ```

---

**Constructor Summary**

`EMResultFactory()`


| **Method Summary** | |
| --- | --- |
| `static EMResult` | `read(java.io.BufferedReader buffer)` |
| `static void` | `write(EMResult result, java.io.OutputStream stream)` |
| `static void` | `write(EMResult result, java.io.PrintWriter writer)` |

**Methods inherited from class java.lang.Object**

`clone, equals, finalize, getClass, hashCode, notify, notifyAll, toString, wait, wait, wait`

**Constructor Detail**

### EMResultFactory

```
public EMResultFactory()
```


**Method Detail**

### read

```
public static EMResult read(java.io.BufferedReader buffer)
                     throws java.io.IOException
```

:   **Throws:**: `java.io.IOException`

---


### write

```
public static void write(EMResult result,
                         java.io.OutputStream stream)
                  throws java.io.IOException
```

:   **Throws:**: `java.io.IOException`

---


### write

```
public static void write(EMResult result,
                         java.io.PrintWriter writer)
```

:   **Parameters:**: `result` -: `writer` -


---


|  |  |  |
| --- | --- | --- |
| **Overview** \| **Package** \| **Class** \| **Use** \| **Tree** \| **Deprecated** \| **Index** \| **Help** | |  |
| **PREV CLASS**   **NEXT CLASS** | **FRAMES**    **NO FRAMES**     **All Classes** |  |
| SUMMARY: NESTED \| FIELD \| CONSTR \| METHOD | DETAIL: FIELD \| CONSTR \| METHOD |  |


---
